# Around the Hospitals

**Published:** 1894-01-27

**Authors:** 


### Around the Hospitals.

Notice to the Managers of Institutions.—Special space is now reserved for the insertion of notices of hospital meetings and festivals. The Editor requests that all notices may reach The Hospital Office, 428, Strand, at least one week before the date of each meeting.

**The Kilmarnock Infirmary.**—There are visible signs of life about the work of the Kilmarnock Infirmary. Indeed the annual record is a very interesting one, when once the trouble is overcome incurred by perusing a most indifferently arranged report. Taking this up after studying the annals of another institution, evidently compiled to attract and not fatigue attention, the task of gathering that patients were increasing, legacies falling in, and collections prospering, was by no means an easy one. The burying of so much that is encouraging and admirable in wretchedly small and closely set type is certainly like hiding one's light under a bushel. Events of the year have been stirring ones at Kilmarnock. An example of promptness is given which might usefully be studied by dilatory authorities. Typhoid appeared at Kilmarnock and its neighbourhood. The infirmary authorities forseeing the strain likely to be placed on the accommodation promptly set to work, and within four weeks an emergency ward was ready for occupation. This additional provision proved barely sufficient, and the matron and nurses earned much praise for the way they met the severe pressure on their labours. The new ward was erected at a cost of £550. The inside walls are of enamelled brick. The institution is evidently appreciated in the neighbourhood. A bazaar organized to clear a building debt of £2,000 realised no less than £3,657. In the same year Lady Howard de Walden endowed a cot in the children's ward, presenting £1,200 for the purpose, and many other gifts and legacies also fell to the lot of the infirmary.

**Lincoln County Hospital.**—The annual report of this institution shows that at the present time a balance of £83 is due to the treasurer, yet the financial position of the institution is not serious, seeing that the receipts only fell short of the expenditure during the past year by £60 5s. 1d., and this amount, with the adverse balance of £22 15s. with which they commenced the year, accounted for the balance of £83 against it. Some six or seven years ago almost every year the increase fell short of the expenditure by £600 or £700, and therefore the present financial position of the hospital when compared with its position several years ago shows a decided improvement. The improvement had not been confined to one year, but now extended over a period of several years, and provided there was no relaxation in the efforts of those who worked so well and with such great success for the institution there is no reason why that improvement should not be maintained.

